# High NCALD expression predicts poor prognosis of cytogenetic normal acute myeloid leukemia

**DOI:** 10.1186/s12967-019-1904-5

**Published:** 2019-05-20

**Authors:** Ying Song, Weilong Zhang, Xue He, Xiaoni Liu, Ping Yang, Jing Wang, Kai Hu, Weiyou Liu, Xiuru Zhang, Hongmei Jing, Xiaoliang Yuan

**Affiliations:** 1 The First Clinical College of Gannan Medical University, Ganzhou, 341000 China; 2 Department of Hematology, Lymphoma Research Center, Peking University Third Hospital, No. 49 North Garden Road, Haidian District, Beijing, 100191 People’s Republic of China; 3 Department of Pathology, Beijing Tiantan Hospital Affiliated With Capital Medical University, No. 6 Tiantan Xili, Beijing, 100050 China; 4 Department of Respiratory Medicine, The First Affiliated Hospital of Gannan Medical University, No. 23 Qingnian Road, Zhanggong District, Ganzhou, 341000 China

**Keywords:** NCALD, Cytogenetic normal acute myeloid leukemia (CN-AML), LSC, Prognostic factor

## Abstract

**Background:**

Acute myeloid leukemia (AML) is a heterogeneous disease in terms of genetic basis, clinical, biological and prognostic, and is a malignant clonal disease of leukemia stem cells (LSCs). Nearly half of adult AML patients exhibit a cytogenetic normal acute myeloid leukemia (CN-AML). The expression level of NCALD gene was associated with the prognosis of ovarian cancer and non-small cell lung cancer (NSCLC). The expression level of NCALD gene is still unclear in the prognosis of patients with AML.

**Method:**

We integrated 5 independent datasets totally 665 AML patients (497 CN-AML patients) to analyzed relation between NCALD gene expression and the clinical FAB classification, gene mutation, therapy, prognosis of CN-AML. We analyzed the NCALD gene expression with the prognosis and LSC of 165 AML patients from The Cancer Genome Atlas (TCGA) dataset and 78 AML patients from GEO dataset.

**Results:**

High NCALD-expressing CN-AML patients were associated with poor event-free survival (EFS) and overall survival (OS) compared to low NCALD expression (EFS, P < 0.0001, OS, P < 0.0001). In AML patients of allogeneic hematopoietic stem cell transplantation (allo-HSCT), high NCALD expression was associated with poor survival prognosis in EFS and OS (EFS, P < 0.0051, OS, P = 0.028). Post-chemotherapy in AML patients, high NCALD expression led a worse prognosis in EFS and OS (EFS, P = 0.011; OS, P = 0.0056). In multivariate analysis, high NCALD expression was an independent prognostic factor that predicts shorter EFS and OS (EFS, P = 3.84E−05, OS, P = 8.53E−05) of CN-AML.

**Conclusion:**

Our results indicate that high expression of NCALD gene is a poor prognostic factor for CN-AML. NCALD can be considered as independent predictors of CN-AML patients and can be used as a biomarker for the prognosis of CN-AML.

**Electronic supplementary material:**

The online version of this article (10.1186/s12967-019-1904-5) contains supplementary material, which is available to authorized users.

## Background

Acute myeloid leukemia (AML) is a heterogeneous disease in terms of genetic basis, clinical, biological and prognostic, and is a malignant clonal disease of leukemia stem cells (LSCs). According to the prognosis, AML patients can be classified based on favorable-risk, intermediate-risk and adverse-risk [1, 2]. AML can achieve 60% to 70% complete response (CR) post-chemotherapy regimens with supportive care, and with long-term survival reaches 25% to 35% [3]. After active treatment in young AML patients, the 5-year survival rate is between 40 and 50%, and there are still treatment challenges in elderly AML patients [3]. However, recurrence is a major obstacle to cure after AML patients achieve CR [4]. The high recurrence rate of AML is considered to be the persistence of LSC [5]. Allogeneic hematopoietic stem cell transplantation (allo-HSCT) is the best treatment for many AML patients [6, 7]. Especially for high-risk AML patients, allo-HSCT can achieve the first complete remission (CR1) and become an effective treatment [8]. As for intermediate-risk acute myeloid leukemia (IR-AML), allo-HSCT is an independent favorable factor for EFS and OS [9].

Nearly half of adult AML patients exhibit a cytogenetic normal acute myeloid leukemia (CN-AML) [10]. CN-AML was classified as IR-AML. Next-generation sequencing detected mutations in CN-AML for 19 AML-related genes, and each patient detected at least one mutation: DNMT3A, IDH2, IDH1, NRAS, NPM1, TET2, ASXL1, PTPN11, and RUNX1 [11]. The prognosis can be further stratified according to different gene mutation combinations in CN-AML patients. For example, mutations in NPM1 and CEBPA are associated with a good prognosis in CN-AML [12, 13], whereas DNMT3, TET2, and RUNX1 mutations are associated with poor prognosis in CN-AML [14–16]. Besides, high expression of genes including ITPR2, MAPKBP1, CPNE3, RUNX1 and ATP1B1 are associated with poor prognosis of CN-AML, while high expression of LEF1 is considered as a favorable prognostic factor for CN-AML [17–22]. Therefore, the identification of new biomarkers can help to predict the prognosis risk of CN-AML.

NCALD (Neurocalcin Delta) is a member of the Neuronal Calcium Sensor family of calcium binding proteins. The expression of NCALD gene is more abundant in the brain, testis, ovary and small intestine [23]. Experiments have shown that NCALD is a potential hippocampal memory-related factor related to obesity [24]. NCALD expression is down-regulated in lung cancer tissues, while low NCALD expression levels are associated with poor prognosis in non-small cell lung cancer (NSCLC) patients [25]. NCALD gene expression was significantly different in survival analysis, indicating that low expression of NCALD gene predicts poor prognosis in patients with ovarian cancer [26]. However, the expression level of NCALD gene in CN-AML patients has never been reported. Thus, we analyzed the relationship between the expression of NCALD and the prognosis of CN-AML.

## Materials and methods

### Data source and gene expression analysis

The method of calculating the probe set measurements for all Gene Expression Omnibus (GEO) (https://www.ncbi.nlm.nih.gov/geo) arrays uses RMA algorithm (robust multiarray averaging). The relative expression values of RNA were log-transformed using log2. Gene expression profiles of The Cancer Genome Atlas (TGCA) (https://portal.gdc.cancer.gov/) AML patients were detected from the RNA-seq data showed with RPKM (Reads of Per Kilobase per Million mapped reads) and further log2 transformed. We calculated the *P* value (and hazard ratio) of each gene expression from the RNA-seq data and EFS/OS of TCGA CN-AML patients using survcomp package. NCALD located in the top position through the rank of the P-value. We divided patients into high-NCALD group and low-NCALD group by NCALD expression levels using maximally selected rank statistics algorithm from survminer package. We also resort to a different method for patient stratification division based on NCALD gene expression quartiles. Top 75% patients are NCALD high expression group and the other 25% patients are NCALD low expression group by ranking NCALD gene expression from high to low. EFS and OS are clinical end points. The time from diagnosis to the first event (last visit or no complete remission or relapse) was defined as EFS. The time from diagnosis to death for any cause or to the last follow-up was defined as OS. The written informed consent of all patients in this study was consistent with the Helsinki Declaration.

We studied totally 165 AML patients from TCGA dataset [27]. Of these, 75 AML patients are CN-AML, 67 patients underwent allo-HSCT and 94 patients received chemotherapy. All the gene expression was detected by RNA-seq method in the TCGA AML patients. Survival prognosis of EFS and OS in AML patients was analyzed by NCALD gene expression.

We studied totally 240 CN-AML patients from the GSE12417 (https://www.ncbi.nlm.nih.gov/geo/query/acc.cgi?acc=GSE12417) [28]. Gene expression was performed by using the Affymetrix Human Genome U133A/B Array and Affymetrix Human Genome U133 Plus 2.0 Array. The relationship between NCALD gene expression and OS prognosis in patients with CN-AML was analyzed.

We studied totally 78 CN-AML patients from the GSE22778 (https://www.ncbi.nlm.nih.gov/geo/query/acc.cgi?acc=GSE22778) [29]. The relationship between NCALD gene expression and OS prognosis of CN-AML patients was analyzed.

We studied totally 104 CN-AML patients from the GSE71014 (https://www.ncbi.nlm.nih.gov/geo/query/acc.cgi?acc=GSE71014) [30]. Gene expression was performed by using the Illumina HumanHT-12 V4.0 expression beadchip. The relationship between NCALD gene expression and OS prognosis CN-AML patients was analyzed.

Gene expression was performed by using the NanoString Expression Assay system in GSE76004 (https://www.ncbi.nlm.nih.gov/geo/query/acc.cgi?acc=GSE76004) [31]. We analyzed the expression of NCALD genes in different LSC subgroups from 78 AML patients.

### Statistical analysis

Statistical analysis of this study was performed using R software v3.1.3 (ggplot2 and survminer packages). Descriptive statistics are used to summarize the clinical and molecular characteristics of the patient. Survival analysis was assessed by log rank test and Kaplan–Meier curves. Comparison of NCALD expression levels between CD34/CD38 subgroups using one-way analysis of variance (Anova test). The expression of NCALD between the LSC- and LSC + groups was determined by unpaired t test. The statistical method for quantitative variables is t test or Anova test. The statistical method for categorical variables is Fisher’s exact test. Univariate and multivariate analysis of EFS and OS in 74 TCGA CN-AML were performed using Cox regression analysis. One TCGA CN-AML patient without the mutation information was not used in the univariate and multivariate Cox regression analysis. Variables of with P < 0.05 in univariate analysis were selected for multivariate analysis. The time dependent ROC curves estimation from EFS and OS were conducted using survival ROC package. In all statistical methods, P value < 0.05 was considered statistically significant.

## Results

### Clinical baseline characteristics of CN-AML

75 TCGA CN-AML patients were divided into NCALD-high group and NCALD-low group, according to NCALD expression level using maximally selected rank statistics algorithm, and the clinical and molecular characteristics between the two groups were compared (Table 1). Patients in the NCALD-high group had more relapses than the NCALD-low group (P = 0.001; Fisher’s exact test). There were no significant differences in basic characteristics between the two groups, such as sex, race, FAB subtypes, WBC, transplant, chemotherapy, genetic mutations (DNMT3A, NPM1, TET2, FLT3, IDH2, IDH1, RUNX1, NRAS, WT1, CEBPA, PTPN11, KRAS), complete response and relapse of before transplantation (all P > 0.05; Fisher’s exact test). Age, bone marrow blast (BM-blast), peripheral blood blast (PB-blast) and WBC also had no significant differences between the two groups (all P > 0.05; unpaired t test).

**Table 1:** Baseline patient characteristics according to the expression level of NCALD in TCGA CN-AML patients

Characteristics	Level	NCALD-low	NCALD-high	P-value
n		20	55	
Sex (%)	Female	8 (40.0)	30 (54.5)	0.305
	Male	12 (60.0)	25 (45.5)	
Race (%)	Black	0 (0.0)	3 (5.5)	0.54
	Others	0 (0.0)	1 (1.8)	
	Unknown	2 (10.0)	11 (20.0)	
	White	18 (90.0)	40 (72.7)	
FAB (%)	M0	0 (0.0)	3(5.5)	0.9
	M1	8 (40.0)	16 (29.1)	
	M2	4 (20.0)	15 (27.3)	
	M4	4 (20.0)	11 (20.0)	
	M5	4 (20.0)	8 (14.5)	
	M7	0 (0.0)	1 (1.8)	
	Unknown	0 (0.0)	1 (1.8)	
Age (mean (sd))		51.15 (15.79)	55.80 (17.47)	0.3
BM_BLAST (mean (sd))		75.45 (15.39)	69.02 (19.16)	0.181
WBC (mean (sd)) × 10^9^/L		56.91 (53.67)	49.00 (57.14)	0.592
PB_BLAST (mean (sd))		48.00 (36.32)	43.36 (32.46)	0.598
Karyotype (%)	Normal	20 (100.0)	55 (100.0)	NA
Risk (%)	Intermediate	20 (100.0)	55 (100.0)	NA
Induction (%)	7 + 3	8 (40.0)	23 (41.8)	0.852
	7 + 3+3	6 (30.0)	13 (23.6)	
	7 + 3+3 + others	2 (10.0)	1 (1.8)	
	7 + 3+others	2 (10.0)	6 (10.9)	
	Cytarabine	0 (0.0)	1 (1.8)	
	Decitabine	2 (10.0)	3 (5.5)	
	Decitabine + others	0 (0.0)	1 (1.8)	
	Hydrea + others	0 (0.0)	1 (1.8)	
	No treatment	0 (0.0)	1 (1.8)	
	Others	0 (0.0)	1 (1.8)	
	Revlimid	0 (0.0)	4 (7.3)	
Transplant (%)	Auto	3 (15.0)	1 (1.8)	0.34
	Chemotherapy	10 (50.0)	27 (49.1)	
	Haplo	0 (0.0)	1 (1.8)	
	MUD	4 (20.0)	12 (21.8)	
	No treatment	0 (0.0)	1 (1.8)	
	sib Allo	3 (15.0)	13 (23.6)	
Before_transplant (%)	CR 1	7 (35.0)	11 (20.0)	0.876
	CR 2	2 (10.0)	5 (9.1)	
	CR 3	0 (0.0)	1 (1.8)	
	No transplant	10 (50.0)	27 (49.1)	
	No treatment	0 (0.0)	1 (1.8)	
	Others	1 (5.0)	7 (12.7)	
	Rel 1	0 (0.0)	2 (3.6)	
	Rel 2	0 (0.0)	1 (1.8)	
Relapse (%)	No	14 (70.0)	14 (25.5)	0.001
	Yes	6 (30.0)	41 (74.5)	
DNMT3A (%)	Mutation	7 (35.0)	22 (40.0)	0.846
	Unknown	0 (0.0)	1 (1.8)	
	WT	13 (65.0)	32 (58.2)	
NPM1 (%)	Mutation	13 (65.0)	30 (54.5)	0.705
	Unknown	0 (0.0)	1 (1.8)	
	WT	7 (35.0)	24 (43.6)	
TET2 (%)	Mutation	4 (20.0)	5 (9.1)	0.443
	Unknown	0 (0.0)	1 (1.8)	
	WT	16 (80.0)	49 (89.1)	
FLT3 (%)	Mutation	7 (35.0)	23 (41.8)	0.71
	Unknown	0 (0.0)	1 (1.8)	
	WT	13 (65.0)	31 (56.4)	
IDH2 (%)	Mutation	1 (5.0)	9 (16.4)	0.464
	Unknown	0 (0.0)	1 (1.8)	
	WT	19 (95.0)	45 (81.8)	
IDH1 (%)	Mutation	4 (20.0)	5 (9.1)	0.443
	Unknown	0 (0.0)	1 (1.8)	
	WT	16 (80.0)	49 (89.1)	
RUNX1 (%)	Mutation	0 (0.0)	7 (12.7)	0.228
	Unknown	0 (0.0)	1 (1.8)	
	WT	20 (100.0)	47 (85.5)	
NRAS (%)	Mutation	3 (15.0)	3 (5.5)	0.512
	Unknown	0 (0.0)	1 (1.8)	
	WT	17 (85.0)	51 (92.7)	
WT1 (%)	Mutation	1 (5.0)	4 (7.3)	1
	Unknown	0 (0.0)	1 (1.8)	
	WT	19 (95.0)	50 (90.9)	
CEBPA (%)	Mutation	4 (20.0)	4 (7.3)	0.326
	Unknown	0 (0.0)	1 (1.8)	
	WT	16 (80.0)	50 (90.9)	
PTPN11 (%)	Mutation	1 (5.0)	4 (7.3)	1
	Unknown	0 (0.0)	1 (1.8)	
	WT	19 (95.0)	50 (90.9)	
KRAS (%)	Mutation	1 (5.0)	2 (3.6)	1
	Unknown	0 (0.0)	1 (1.8)	
	WT	19 (95.0)	52 (94.5)	

n, number of patients; FAB, French–American–British subtypes; BM-blast, bone marrow blast; PB-blast, peripheral blood blast; WBC, white blood cell; MUD, matched unrelated donor; sib-allo HSCT, sib allogeneic stem cell transplantation; Haplo, haploidentical; CR 1, first complete remission; Rel 1, first relapse. Age, BM-blast, WBC and PB-blast statistical methods using unpaired t test, two sided. Use the Fisher’s exact test for statistical methods of categorical variables

CN-AML patients from other GEO datasets (GSE12417, GSE22778) were divided into NCALD-high group and NCALD-low group by the same method. The clinical and molecular characteristics between the two groups were compared. FAB subtypes, sex and gene mutations (NPM1, RUNX1, TET2) were not significantly differences in the basic characteristics between the two groups (Additional file 1: Tables S1–S4, all P > 0.05; Fisher’s exact test), and the age was not significantly difference between the two groups (Additional file 1: Tables S1–S4, all P > 0.05; unpaired t test).

### High NCALD expression predicts worse survival in CN-AML patients

**Fig. 1:**
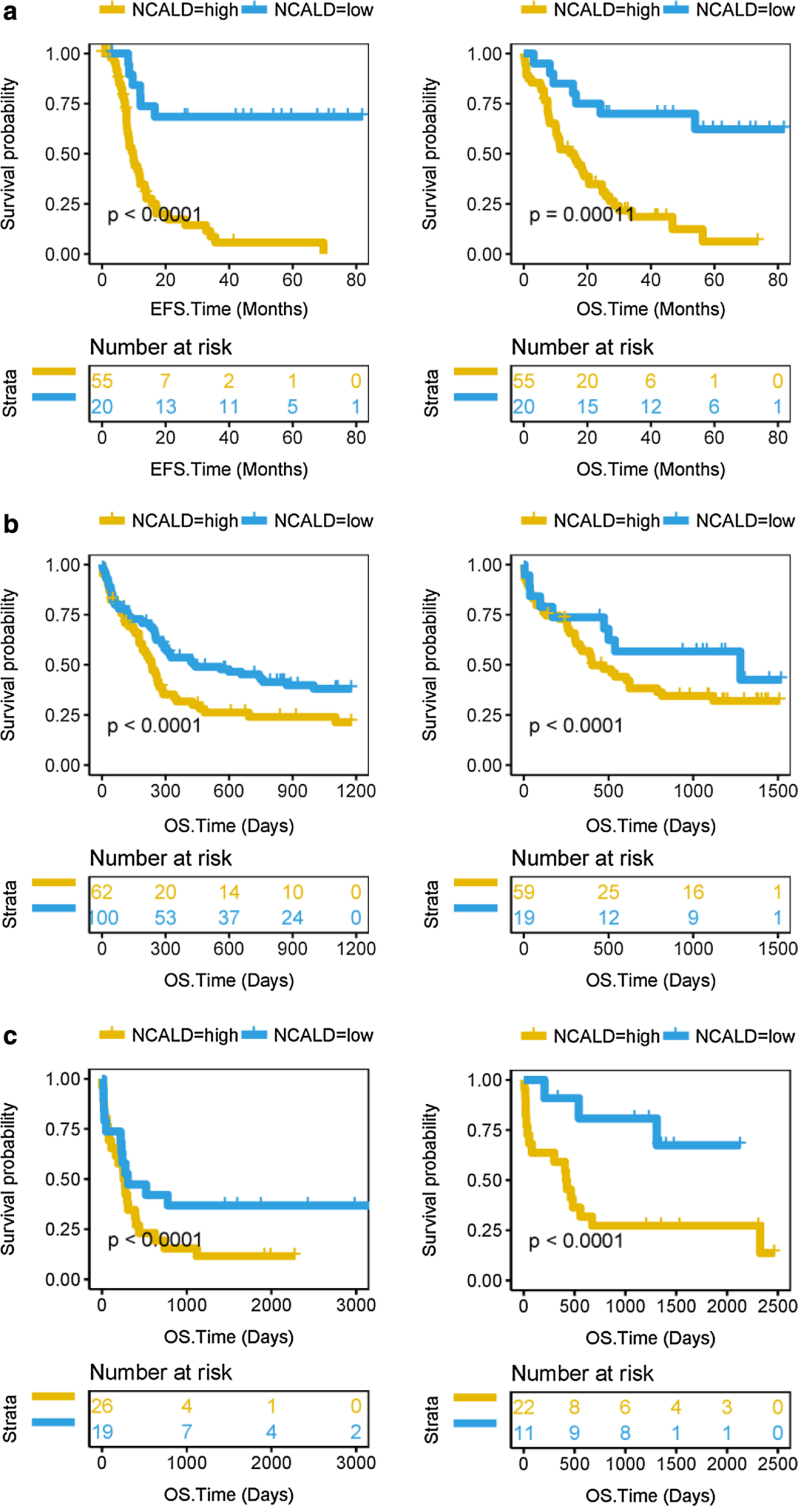
High NCALD expression predicts worse survival of CN-AML. The X axis represents time and the Y axis represents survival probability. A, Kaplan–Meier curves were used for EFS and OS in different NCALD expression groups of CN-AML in the TCGA dataset. The cutoff value for the high and low NCALD groups was 4.74. EFS, P < 0.0001; OS, P = 0.00011; log rank test. B, Kaplan–Meier curves were used for OS in different NCALD expression groups of CN-AML in GSE12417. The cutoff values for the high and low NCALD groups were 8.1794 (left)/5.882 (right). OS, P < 0.0001; log rank test. C, Kaplan–Meier curves were used for OS in different NCALD expression groups in GSE22778. The cutoff values for the high and low NCALD groups were − 1.384 (left)/− 1.095 (right). OS, P < 0.0001; log rank test

We analyzed the gene expression profiles of NCALD of EFS and OS in 75 CN-AML patients from TCGA dataset. There was a significant difference between the NCALD-high group and the NCALD-low group. High expression of NCALD gene in EFS and OS of CN-AML patients has a lower prognosis than low expression NCALD gene (Fig. 1a, EFS, P < 0.0001; OS, P = 0.00011; log rank test). We analyzed the NCALD gene expression profiles of OS in 240 patients with CN-AML from the GSE12417 dataset. Patients with high NCALD expression in CN-AML have a worse prognosis in OS than low NCALD expression (Fig. 1b, OS, P < 0.0001; log rank test). Then, we also found that patients with high NCALD expression had a worse prognosis in OS than low NCALD expression. These patients were 78 CN-AML patients from GSE22778 (Fig. 1c, OS, P < 0.0001; log rank test) and 104 CN-AML patients from GSE71014 (Additional file 2: Figure S1, OS, P < 0.0001; log rank test).

We used a different method for patient classification based on NCALD gene expression quartiles. All datasets showed that patients with high NCALD expression have a poor prognosis (Additional file 2: Figure S2, all P < 0.001; Additional file 2: Figure S3, P < 0.0001, log rank test).

### NCALD expression predicts survival after treatment in AML patients

**Fig. 2:**
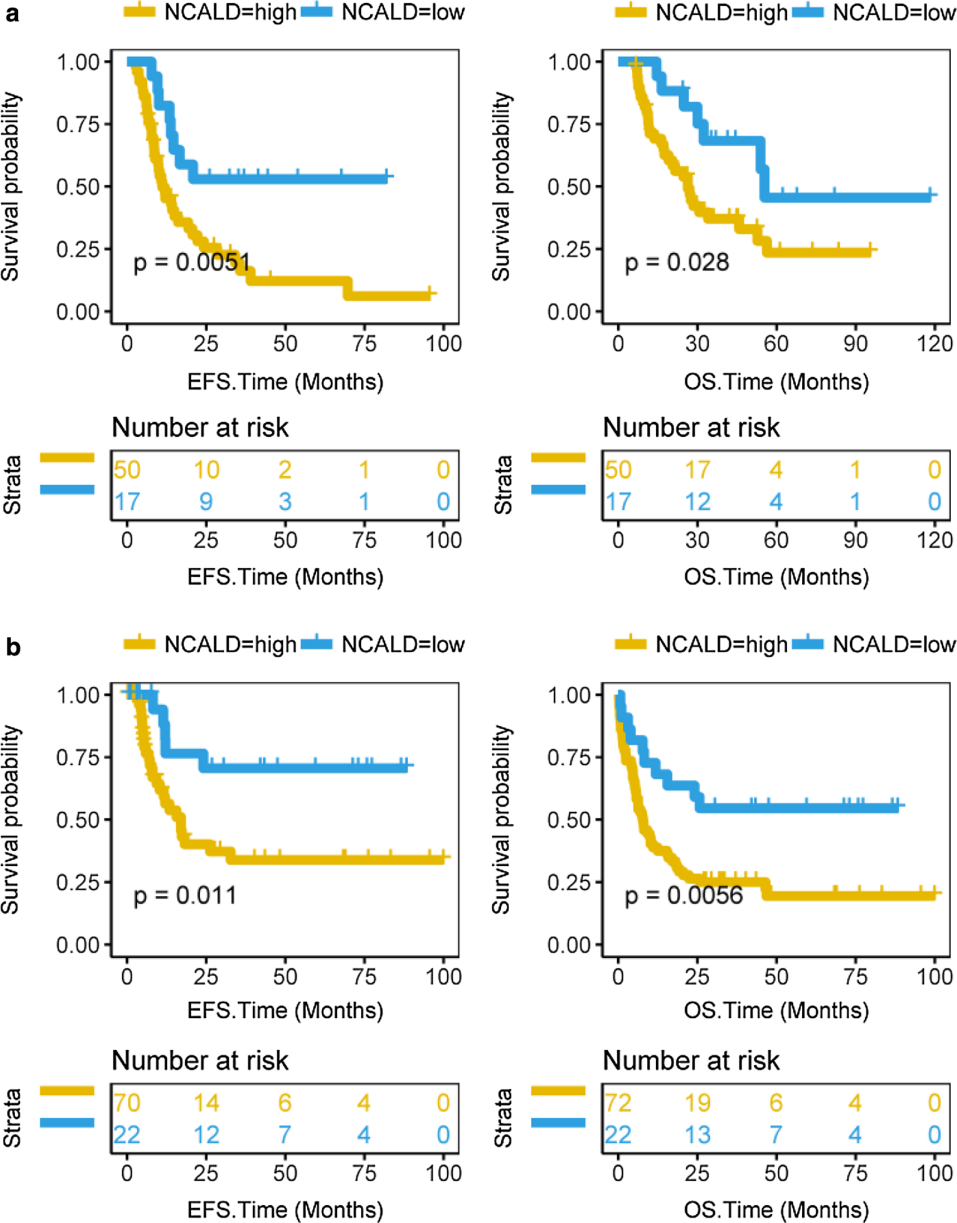
High NCALD expression predicts worse survival of allo-HSCT or post-chemotherapy AML patients from the TCGA dataset. The X axis represents time (months) and the Y axis represents survival probability. A, Kaplan–Meier curves were used of EFS and OS in different NCALD expression groups after allo-HSCT. The cutoff value for the high and low NCALD groups was 5.30. EFS, P = 0.0051; OS, P = 0.028; log rank test. B, Kaplan–Meier curves were used of EFS and OS in different NCALD expression group after chemotherapy. The cutoff value for the high and low NCALD groups was 4.66. EFS, P = 0.011; OS, P = 0.0056; log rank test

To investigate the gene expression of NCALD in AML patients after allo-HSCT or chemotherapy, we examined the gene expression profiles of NCALD in EFS and OS of 67 allo-HSCT patients from TCGA dataset. In EFS and OS, the NCALD-high group was associated with poor survival of allo-HSCT patients, while the NCALD-low group had good survival (Fig. 2a, EFS, P = 0.0051; OS, P = 0.028; log rank test). We additionally analyzed the prognostic value of NCALD gene expression in 94 AML patients after chemotherapy. Over expression of NCALD gene predicts worse EFS and OS in AML patients after chemotherapy from the TCGA dataset (Fig. 2b, EFS, P = 0.011; OS, P = 0.0056; log rank test).

We divided allo-HSCT or post-chemotherapy patients from the TCGA dataset into two groups based on the quartile of NCALD expression levels. The result showed that patients with high NCALD expression have a poor prognosis (Additional file 2: Figure S4, all P < 0.05, log rank test).

### Univariate and multivariate analysis of EFS and OS

Univariate analysis for 17 variables including NCALD, age, WBC count, PB-blast, BM-blast and genes mutation (DNMT3A, NPM1, TET2, FLT3, IDH2, IDH1, RUNX1, NRAS, WT1, CEBPA, PTPN11, KRAS, mutation vs. wild type) from 74 TCGA CN-AML patients was conducted (Additional file 1: Table S5). Univariate analysis showed that FLT3 mutations are associated with shorter EFS (P = 0.0214; Cox regression), while high NCALD expression and DNMT3A mutations contributed to worse EFS and OS (NCALD, EFS, P = 3.90E−05, OS, P = 0.000115; DNMT3A, EFS, P = 0.0193, OS, P = 0.0402; Cox regression). NCALD, DNMT3A, and FLT3 have meaningful P values (P < 0.05) and HRs > 1 in EFS, while NCALD and DNMT3A have meaningful P values (P < 0.05) in OS and their HRs > 1 (Additional file 2: Figure S5). Among them, the NCALD gene is the most significant predictor of CN-AML survival (Additional file 2: Figure S5).

We selected NCALD expression levels and genes mutation (DNMT3A, FLT3, mutation vs. wild type) for multivariate analysis of EFS and OS (Table 2). Multivariate analysis assessed independent risk factors for clinical prognostic of EFS and OS in CN-AML. High NCALD expression and DNMT3A mutations were significantly associated with EFS (NCALD, P = 3.84E−05; DNMT3A, P = 0.0106; Cox regression). High NCALD expression and mutation of DNMT3A were significantly associated with OS (NCALD, P = 8.53E−05; DNMT3A, P = 0.0243; Cox regression).

**Table 2:** Multivariate analysis for EFS and OS of TCGA CN-AML patients

Variables	HR	95% CI for HR		*P*-value
		Lower	Upper	
EFS				
DNMT3A	2.241	1.2064	4.161	0.0106
FLT3	1.66	0.9175	3.003	0.0939
NCALD	1.667	1.3068	2.125	3.84E−05
OS				
DNMT3A	1.937	1.09	3.445	0.0243
NCALD	1.618	1.273	2.056	8.53E−05

EFS, event-free survival time; OS, overall survival time; HR, hazard ratio; CI, confidence interval. Linear variable: NCALD (NCALD gene expression log2). Binary variable: DNMT3A, FLT3 (mutation vs. wild type)

### NCALD is a potential marker for predicting prognosis

**Fig. 3:**
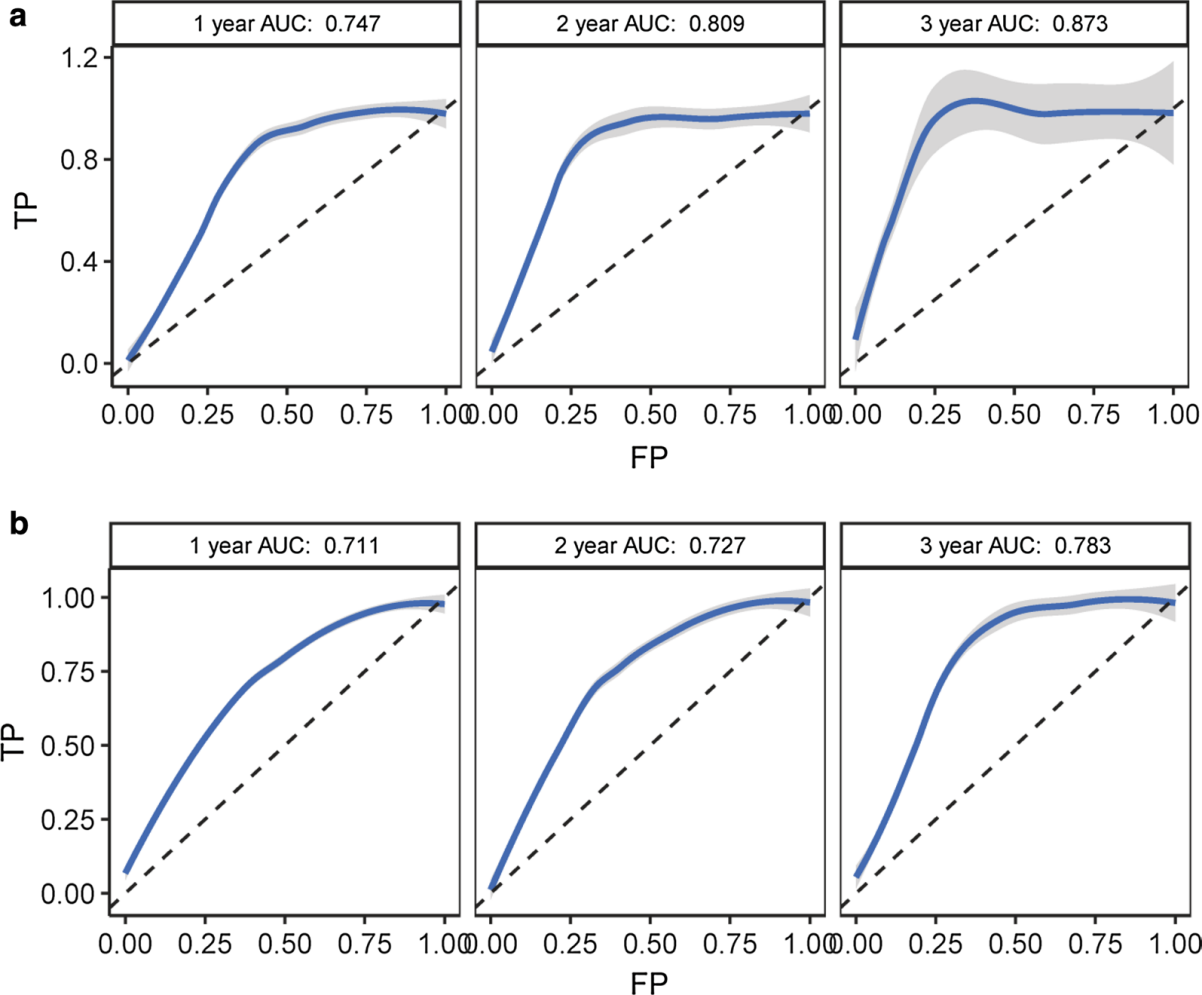
ROC curves of survival for NCALD gene expression in TCGA CN-AML patients. The X axis represents false positive (FP) and the Y axis represents true positive (TP). A, ROC curves for NCALD expression and EFS of 75 TCGA CN-AML patients were performed. AUC reached 0.747 (1 year survival, left panel), 0.809 (2 year survival, middle panel) and 0.873 (3 year survival, right panel) respectively. B, ROC curves for NCALD expression and OS of 75 TCGA CN-AML patients were performed. AUC reached 0.711 (1 year survival, left panel), 0.727 (2 year survival, middle panel) and 0.783 (3 year survival, right panel) respectively. The grey shadow of the curves represents 95% confidence interval

To further investigate the potential clinical value of NCALD, we performed an analysis of the area under the receiver operating characteristic curve (AUC-ROC) of the survival model for variables from TCGA CN-AML. In the ROC curve analysis of age (Additional file 2: Figure S6), WBC (Additional file 2: Figure S7), DNMT3A (Additional file 2: Figure S8), and NCALD (Fig. 3), NCALD expression reached the largest area under the curve (AUC) of both EFS and OS. These results suggest that NCALD seems to be an excellent predictor of CN-AML prognosis.

### Expression level of NCALD in LSC

Stem cells are divided into 4 cell populations based on fractions of the CD34 and CD38 phenotypes. The expression of NCALD was different in each subgroups (Additional file 2: Figure S9a, P = 0.00019; Anova test). Compared with the mean of all subgroups, the expression of NCALD gene was reduced in the CD34/CD38−/ −group (Additional file 2: Figure S9a, P ≤ 0.001; unpaired t test, two sided), and the expression of CD34/CD38 ± group was increased (Additional file 2: Figure S9a, P ≤ 0.01; unpaired t test, two sided), while the CD34/CD38∓ group and CD34/CD38 +/+ group showed no significant difference (Additional file 2: Figure S9a, P > 0.05; unpaired t test, two sided). To investigate the expression of NCALD gene in LSC, we analyzed 78 AML patients from the GSE76004 dataset. The data showed that the expression of NCALD gene in LSC + group was higher than it in LSC-group (Additional file 2: Figure S9b, P = 0.01; unpaired t test, two sided).

## Discussion

The NCALD gene is a regulator of G-protein coupled receptor signaling, and there are several alternatively spliced gene variants that all encode the same protein. So far, little has been reported known about the relationship between NCALD and cancer, and the prognostic relationship between this gene and CN-AML has not been reported. We analyzed the gene expression of NCALD from AML patients in 5 independent datasets. Our study reports that the expression of NCALD is associated with the prognosis of CN-AML and is an independent risk factor of CN-AML.

Previous studies have reported that low expression of the NCALD gene is associated with the prognosis of some kinds of cancer. NCALD expression is down-regulated in the poor prognosis group of advanced ovarian cancer which suggests that NCALD is a prognostic biomarker for these ovarian cancer patients [32]. Low NCALD expression levels predict poor prognosis in patients with non-small cell lung cancer (NSCLC) [25]. However, in our survival analysis study, we found that the high expression of NCALD of CN-AML patients has a lower prognosis than patients with low NCALD expression. These results suggest that NCALD can predict different prognosis in different cancers and may play a different role in each type of human cancers.

Predicting the prognosis of AML after allo-HSCT treatment is particularly clinically important. Recent studies have shown that high expression of GAS6-mRNA is associated with poor prognosis of allo-HSCT in AML patients [33]. Previous studies of TMEM18, TP53, IDH and DNMT3A are potential biomarkers for AML [34–36]. However, there are still challenges to the prediction of the prognosis of AML, especially the prediction of the prognosis of AML after allo-HSCT. Our results suggest that high NCALD expression in AML patients after allo-HSCT can still predict poor prognosis. High NCALD expression also predicts poor prognosis in AML patients after chemotherapy. Therefore, NCALD may be a biomarker for predicting the prognosis of AML with or without allo-HSCT.

In univariate and multivariate analyses, we found that the DNMT3A mutations are associated with EFS and OS in CN-AML, and that the FLT3 mutations are associated with EFS in CN-AML. This is consistent with previous findings that DNMT3A mutations are associated with shorter EFS and OS [37–39]. Multivariate analysis showed that high expression of NCALD in CN-AML patients was confirmed to be associated with worse EFS and OS. Furthermore, in the ROC curve survival analysis, we found that the relation between NCALD gene expression levels and the prognosis of CN-AML patients was superior to other variables. These results suggest that NCALD should be an independent predictor of prognosis in patients with CN-AML.

The LSC immunophenotypic analysis of all subtypes of AML is CD34+ CD38− [40, 41]. Most of CD34+ and a few of CD34- fractions contain LSC, and LSC was detected in all CD34/CD38 phenotype fractions [31, 42]. AML is initiated by a small fraction of LSCs that maintain extensive proliferation and unrestricted self-renewal of leukemia mother cells, leading to chemoresistance and relapse [43, 44]. High expression of LSC gene is independently associated with poor prognosis in patients with AML [45]. In our study, more LSC+ was present in AML patients with high NCALD gene expression, suggesting that the NCALD gene is enriched in stem cell expression. This result suggests that the NCALD gene may affect LSC.

However, we did not further study the specific function of NCALD in the AML pathogenic signaling pathway. This study only explored the expression of individual genes, and future studies combining different types of potential biomarkers can be conducted to further predict the prognosis of patients with AML.

## Conclusions

In summary, our results indicate that high expression of NCALD gene is a poor prognostic factor for CN-AML. NCALD can be considered as independent predictors of CN-AML patients and can be used as a biomarker for the prognosis of CN-AML.

## Additional files

**Additional file 1.** Additional tables.

**Additional file 2: Figure S1.** High NCALD expression predicts worse survival of CN-AML. The X axis represents time (months) and the Y axis represents survival probability. Kaplan-Meier curves were used for OS in different NCALD expression groups of CN-AML patients from GSE71014. The cutoff values for the high and low NCALD groups were 6.8281. OS, P < 0.0001; log rank test. **Figure S2.** High NCALD expression predicts poor survival of CN-AML. The X axis represents time and the Y axis represents survival probability. All patients were divided into two groups based on quartiles of NCALD expression levels. Top 75% patients are NCALD high expression group and the other 25% patients are NCALD low expression group by ranking NCALD gene expression from high to low. A, Kaplan-Meier curves were used for EFS and OS in different NCALD expression groups of CN-AML in the TCGA dataset. EFS, P < 0.0001; OS, P = 0.00035; log rank test. B, Kaplan-Meier curves were used for OS in different NCALD expression groups of CN-AML in GSE12417. OS, P < 0.0001; log rank test. C, Kaplan-Meier curves were used for OS in different NCALD expression groups in GSE22778. OS, P < 0.0001; log rank test. **Figure S3.** High NCALD expression predicts poor survival of CN-AML. The X axis represents time (months) and the Y axis represents survival probability. All patients were divided into two groups based on quartiles of NCALD expression levels. Top 75% patients are NCALD high expression group and the other 25% patients are NCALD low expression group by ranking NCALD gene expression from high to low. Kaplan-Meier curves were used for OS in different NCALD expression groups of CN-AML patients from GSE71014. OS, P < 0.0001; log rank test. **Figure S4.** High NCALD expression predicts poor survival of AML patients after allo-HSCT or chemotherapy from the TCGA dataset. The X axis represents time (months) and the Y axis represents survival probability. All patients were divided into two groups based on quartiles of NCALD expression levels. Top 75% patients are NCALD high expression group and the other 25% patients are NCALD low expression group by ranking NCALD gene expression from high to low. A, Kaplan–Meier curves were used of EFS and OS in different NCALD expression groups after allogeneic hematopoietic stem cell transplantation (allo-HSCT). EFS, P = 0.0051; OS, P = 0.028; log rank test. B, Kaplan–Meier curves were used of EFS and OS in different NCALD expression group after chemotherapy. EFS, P = 0.011; OS, P = 0.01; log rank test. **Figure S5.** The NCALD gene is the most significant predictor of TCGA CN-AML survivals. The X axis represents the hazard ratio (log2 HR) and the Y axis represents the P-value (−log10). A, HR and P-value scatter plots for EFS univariate analysis of all variables (17 variables). The lower and upper 95% confidence interval of HR was showed in the scatter plot. B, HR and P-value scatter plots for OS univariate analysis of all variables (17 variables). The lower and upper 95% confidence interval of HR was showed in the scatter plot. **Figure S6.** ROC curves of survival for age in TCGA CN-AML patients. The X axis represents false positive (FP) and the Y axis represents true positive (TP). A, ROC curves for age and EFS of 75 TCGA CN-AML patients were performed. AUC reached 0.559 (1 year survival, left panel), 0.551 (2 year survival, middle panel) and 0.591 (3 year survival, right panel) respectively. B, ROC curves for age and OS of 75 TCGA CN-AML patients were performed. AUC reached 0.605 (1 year survival, left panel), 0.62 (2 year survival, middle panel) and 0.605 (3 year survival, right panel) respectively. The grey shadow of the curves represents 95% confidence interval. **Figure S7.** ROC curves of survival for WBC count in TCGA CN-AML patients. The X axis represents false positive (FP) and the Y axis represents true positive (TP). A, ROC curves for WBC count and EFS of 75 CN-AML TCGA patients were performed. AUC reached 0.598 (1 year survival, left panel), 0.633 (2 year survival, middle panel) and 0.604 (3 year survival, right panel) respectively. B, ROC curves for WBC count and OS of 75 CN-AML TCGA patients were performed. AUC reached 0.56 (1 year survival, left panel), 0.624 (2 year survival, middle panel) and 0.616 (3 year survival, right panel) respectively. The grey shadow of the curves represents 95% confidence interval. **Figure S8.** ROC curves of survival for DNMT3A mutation in TCGA CN-AML patients. The X axis represents false positive (FP) and the Y axis represents true positive (TP). A, ROC curves for DNMT3A mutation and EFS of 75 CN-AML TCGA patients were performed. AUC reached 0.639 (1 year survival, left panel), 0.64 (2 year survival, middle panel) and 0.643 (3 year survival, right panel) respectively. B, ROC curves for DNMT3A mutation and OS of 75 CN-AML TCGA patients were performed. AUC reached 0.641 (1 year survival, left panel), 0.599 (2 year survival, middle panel) and 0.622 (3 year survival, right panel) respectively. The grey shadow of the curves represents 95% confidence interval. **Figure S9.** Expression level of NCALD gene in different subtypes of AML. The X axis represents different subtypes and the Y axis represents gene expression. NCALD gene expression was measured as log2. A, Comparison of NCALD expression levels in four subtypes of AML, P = 0.00019; ANOVA, ns, ** and *** indicate P > 0.05, P ≤ 0.01 and P ≤ 0.001, respectively. Each group is compared to the average of the entire data. Add a horizontal dashed line at the average. B, High expression level of NCALD gene in LSC+, P = 0.01; unpaired t test, two sided.

## Data Availability

Please contact the author to get the datasets.

## Abbreviations

AML: acute myeloid leukemia
LSC: leukemia stem cell
CN-AML: cytogenetic normal acute myeloid leukemia
NSCLC: non-small cell lung cancer
TCGA: The Cancer Genome Atlas
EFS: event-free survival
OS: overall survival
FAB: French–American–British subtypes
BM-blast: bone marrow blast
PB-blast: peripheral blood blast
WBC: white blood cell
MUD: matched unrelated donor
allo-HSCT: allogeneic hematopoietic stem cell transplantation
CR: complete response
CR1: first complete remission
IR-AML: intermediate-risk acute myeloid leukemia
NCALD: Neurocalcin Delta
GEO: Gene Expression Omnibus
RPKM: Reads of Per Kilobase per Million mapped reads
ROC: receiver operating characteristic
AUC: area under the curve
