# Supplementary material for: High NCALD expression predicts poor prognosis of cytogenetic normal acute myeloid leukemia
Source: J Transl Med. 2019 May 20;17:166. doi: 10.1186/s12967-019-1904-5 (PMC6528257; doi:10.1186/s12967-019-1904-5)
Supplement: Supplementary file 1 — Additional file 1. Additional tables. [file 12967_2019_1904_MOESM1_ESM.docx]

Additional Table S1. Baseline patient characteristics according to the expression level of NCALD in GSE12417 (U133A) 162 CN-AML patients.

| Characteristics | Level | NCALD-low | NCALD-high | P-value |
| --- | --- | --- | --- | --- |
| n |  | 100 | 62 |  |
| FAB (%) | M0 | 4 ( 4.0) | 1 ( 1.6) | 0.294 |
|  | M1 | 23 (23.0) | 22 (35.5) |  |
|  | M2 | 27 (27.0) | 18 (29.0) |  |
|  | M4 | 26 (26.0) | 16 (25.8) |  |
|  | M5 | 15 (15.0) | 4 ( 6.5) |  |
|  | M6 | 5 ( 5.0) | 1 ( 1.6) |  |
| Age (mean (sd)) |  | 54.17 (15.07) | 57.98 (14.37) | 0.113 |

n, number of patients; FAB, French–American–British subtypes; Age statistical methods using unpaired t test, two sided. Use the Fisher's exact test for statistical methods of categorical variables.

Additional Table S2. Baseline patient characteristics according to the expression level of NCALD in GSE12417 (U133 plus) 78 CN-AML patients.

| Characteristics | Level | NCALD-low | NCALD-high | P-value |
| --- | --- | --- | --- | --- |
| n |  | 19 | 59 |  |
| FAB (%) | M0 | 0 ( 0.0) | 1 ( 1.7) | 0.681 |
|  | M1 | 5 (26.3) | 18 (30.5) |  |
|  | M2 | 11 (57.9) | 23 (39.0) |  |
|  | M4 | 1 ( 5.3) | 10 (16.9) |  |
|  | M5 | 1 ( 5.3) | 5 ( 8.5) |  |
|  | M6 | 1 ( 5.3) | 2 ( 3.4) |  |
| Age (mean (sd)) |  | 57.42 (13.55) | 60.54 (14.27) | 0.404 |

n, number of patients; FAB, French–American–British subtypes; Age statistical methods using unpaired t test, two sided. Use the Fisher's exact test for statistical methods of categorical variables.

Additional Table S3. Baseline patient characteristics according to the expression level of NCALD in GSE22778 (GPL8653) 45 CN-AML patients.

| Characteristics | Level | NCALD-low | NCALD-high | P-value |
| --- | --- | --- | --- | --- |
| n |  | 19 | 26 |  |
| Sex (%) | Female | 11 (57.9) | 18 (69.2) | 0.534 |
|  | Male | 8 (42.1) | 8 (30.8) |  |
| Age (mean (sd)) |  | 52.54 (15.74) | 53.74 (13.42) | 0.785 |
| NPM1 (%) | Mutation | 2 (10.5) | 9 (34.6) | 0.155 |
|  | Unknow | 14 (73.7) | 13 (50.0) |  |
|  | WT | 3 (15.8) | 4 (15.4) |  |
| RUNX1 (%) | Unknow | 13 (68.4) | 11 (42.3) | 0.131 |
|  | WT | 6 (31.6) | 15 (57.7) |  |
| TET2 (%) | Mutation | 2 (10.5) | 3 (11.5) | 0.238 |
|  | Unknow | 10 (52.6) | 7 (26.9) |  |
|  | WT | 7 (36.8) | 16 (61.5) |  |

n, number of patients; Age statistical methods using unpaired t test, two sided. Use the Fisher's exact test for statistical methods of categorical variables.

Additional Table S4. Baseline patient characteristics according to the expression level of NCALD in GSE22778 (GPL10107) 33 CN-AML patients.

| Characteristics | Level | NCALD-low | NCALD-high | P-value |
| --- | --- | --- | --- | --- |
| n |  | 11 | 22 |  |
| Sex (%) | female | 7 (63.6) | 9 (40.9) | 0.282 |
|  | male | 4 (36.4) | 13 (59.1) |  |
| Age (mean (sd)) |  | 45.67 (10.02) | 53.11 (11.36) | 0.075 |
| RUNX1 (%) | Mutation | 1 ( 9.1) | 2 ( 9.1) | 0.366 |
|  | Unknow | 3 (27.3) | 12 (54.5) |  |
|  | WT | 7 (63.6) | 8 (36.4) |  |
| TET2 (%) | Mutation | 2 (18.2) | 2 ( 9.1) | 0.26 |
|  | Unknow | 0 ( 0.0) | 5 (22.7) |  |
|  | WT | 9 (81.8) | 15 (68.2) |  |

n, number of patients; Age statistical methods using unpaired t test, two sided. Use the Fisher's exact test for statistical methods of categorical variables.

Additional Table S5. Univariate analysis for EFS and OS of TCGA CN-AML patients.

| Variables | EFS | | | |  | OS | | | |
| --- | --- | --- | --- | --- | --- | --- | --- | --- | --- |
|  | HR | Lower 95% | Upper 95% | P-value |  | HR | Lower 95% | Upper 95% | P-value |
| Age | 1.01 | 0.991 | 1.03 | 0.305 |  | 1.02 | 1 | 1.04 | 0.0538 |
| BM_BLAST | 0.993 | 0.978 | 1.01 | 0.409 |  | 0.993 | 0.978 | 1.01 | 0.335 |
| WBC | 1 | 0.999 | 1.01 | 0.139 |  | 1 | 1 | 1.01 | 0.0565 |
| PB_BLAST | 1 | 0.994 | 1.01 | 0.557 |  | 0.999 | 0.991 | 1.01 | 0.823 |
| DNMT3A | 2.04 | 1.12 | 3.71 | 0.0193 |  | 1.81 | 1.03 | 3.18 | 0.0402 |
| NPM1 | 0.82 | 0.459 | 1.46 | 0.501 |  | 1.05 | 0.597 | 1.84 | 0.871 |
| TET2 | 0.847 | 0.378 | 1.9 | 0.686 |  | 0.446 | 0.16 | 1.24 | 0.122 |
| FLT3 | 2 | 1.11 | 3.61 | 0.0214 |  | 1.35 | 0.762 | 2.37 | 0.306 |
| IDH2 | 1.03 | 0.457 | 2.31 | 0.947 |  | 0.991 | 0.444 | 2.21 | 0.983 |
| IDH1 | 0.453 | 0.162 | 1.27 | 0.132 |  | 0.422 | 0.151 | 1.18 | 0.101 |
| RUNX1 | 1.52 | 0.598 | 3.86 | 0.379 |  | 1.29 | 0.506 | 3.27 | 0.598 |
| NRAS | 0.446 | 0.137 | 1.45 | 0.178 |  | 0.327 | 0.0793 | 1.35 | 0.122 |
| WT1 | 1.61 | 0.57 | 4.54 | 0.369 |  | 1.07 | 0.385 | 3 | 0.891 |
| CEBPA | 1.25 | 0.528 | 2.97 | 0.611 |  | 1.01 | 0.429 | 2.37 | 0.986 |
| PTPN11 | 0.834 | 0.258 | 2.7 | 0.762 |  | 1.12 | 0.402 | 3.12 | 0.828 |
| KRAS | 2.48 | 0.756 | 8.16 | 0.134 |  | 1.52 | 0.471 | 4.91 | 0.483 |
| NCALD | 1.62 | 1.29 | 2.04 | 3.90E-05 |  | 1.58 | 1.25 | 1.99 | 0.000115 |

EFS, event-free survival time, the time from diagnosis to the first event (last visit or no complete remission or relapse) was defined as EFS; OS, overall survival time. The time from diagnosis to death for any cause or to the last follow-up was defined as OS. HR, hazard ratio; CI, confidence interval. Linear variables: age, WBC, BM, PB blasts and NCALD expression. Age: year; WBC: white blood cell, 10^9^/L; BM-blast, bone marrow blast, %; PB-blast, peripheral blood blast, %. Categorical variables: gene mutations (DNMT3A, NPM1, TET2, FLT3, IDH2, IDH1, RUNX1, NRAS, WT1, CEBPA, PTPN11, KRAS, mutation vs. wild type).
