# Supplementary material for: High NCALD expression predicts poor prognosis of cytogenetic normal acute myeloid leukemia
Source: J Transl Med. 2019 May 20;17:166. doi: 10.1186/s12967-019-1904-5 (PMC6528257; doi:10.1186/s12967-019-1904-5)

Additional file 2: Figure S1

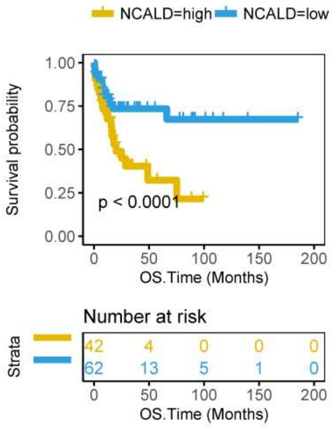

Additional file 2: Figure S2

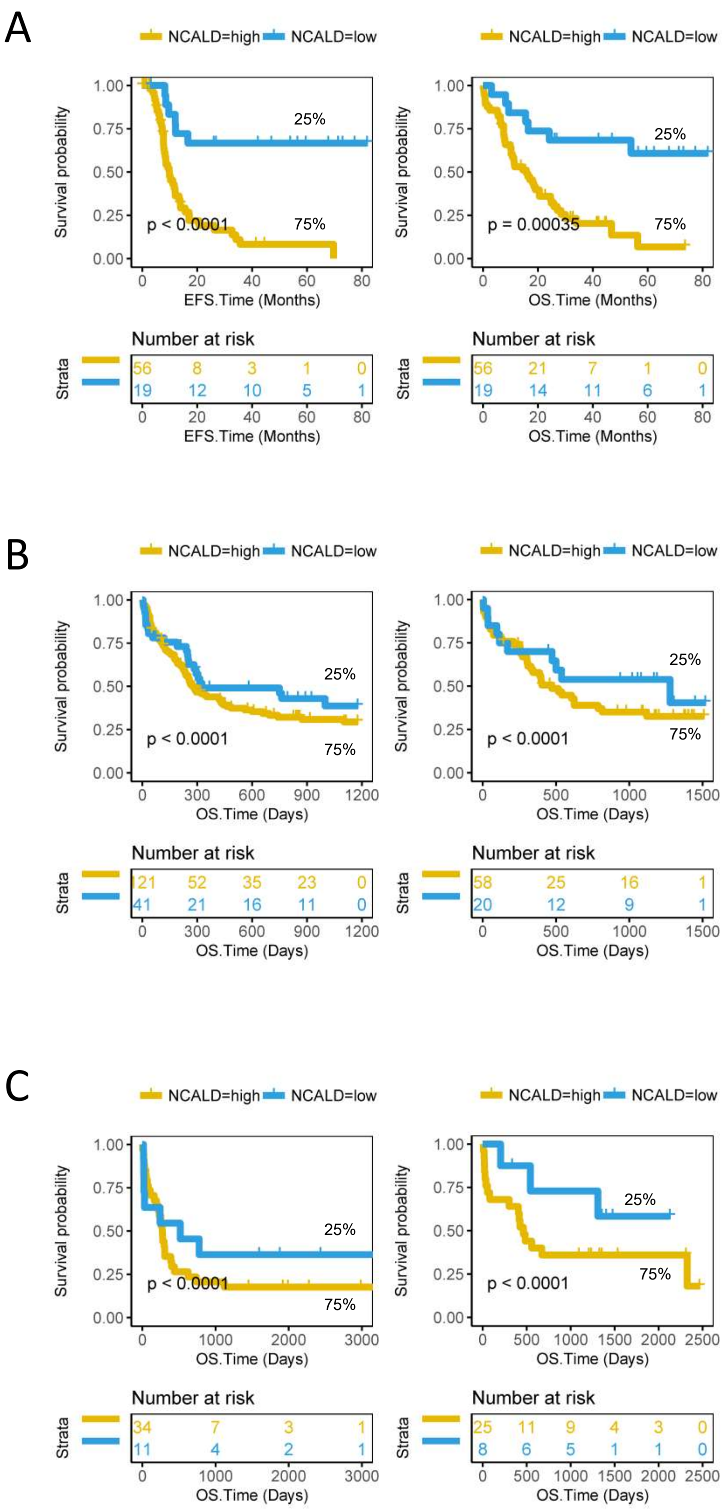

Additional file 2: Figure S3

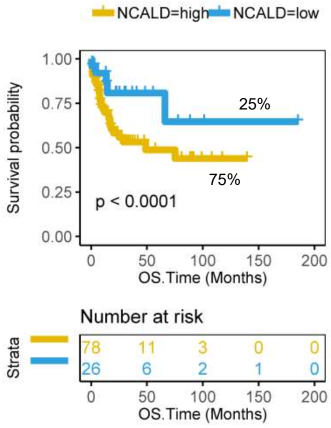

Additional file 2: Figure S4

A

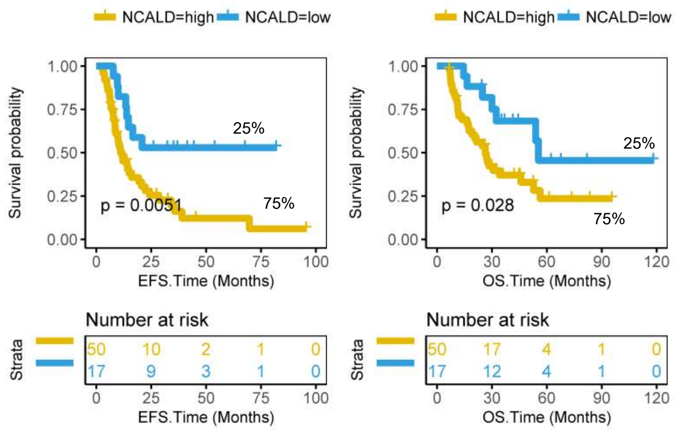

B

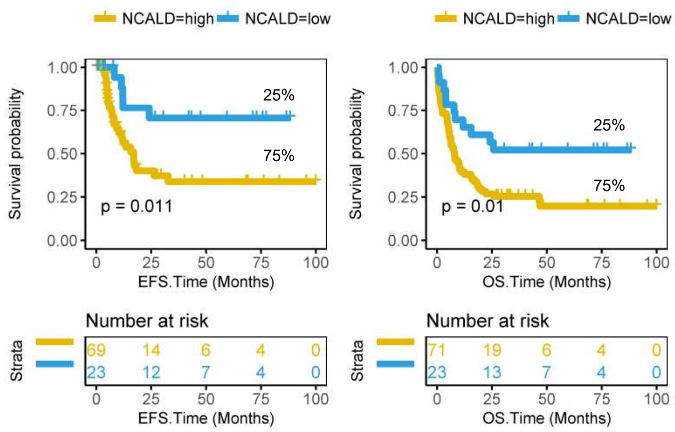

Additional file 2: Figure S5

A

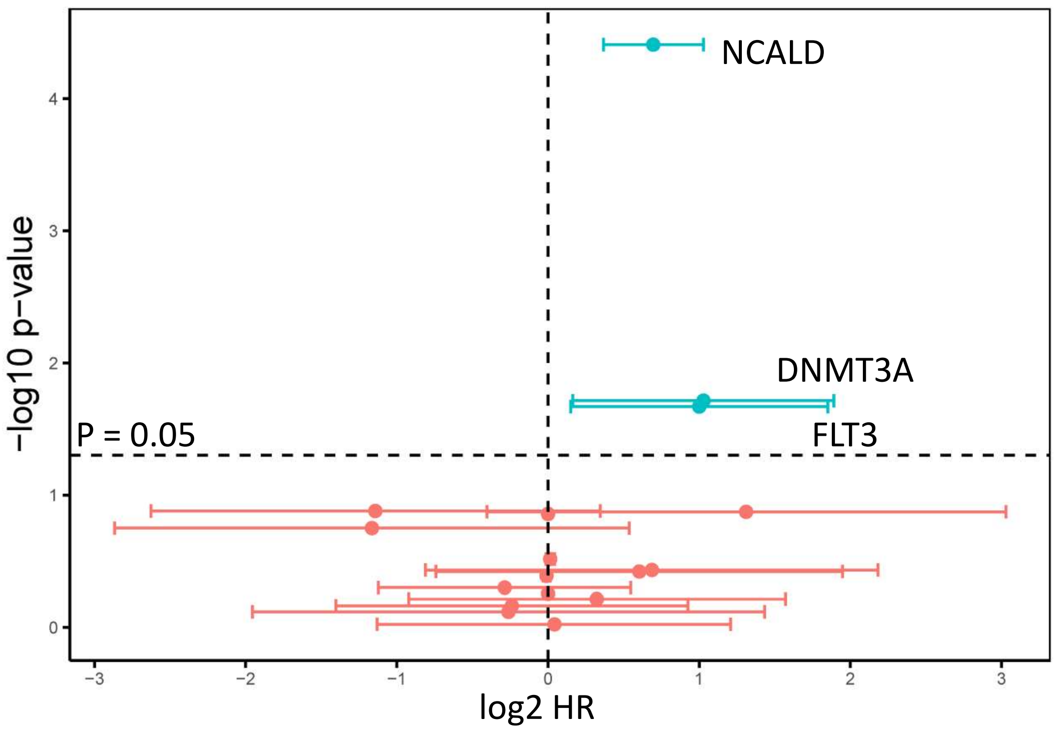

B

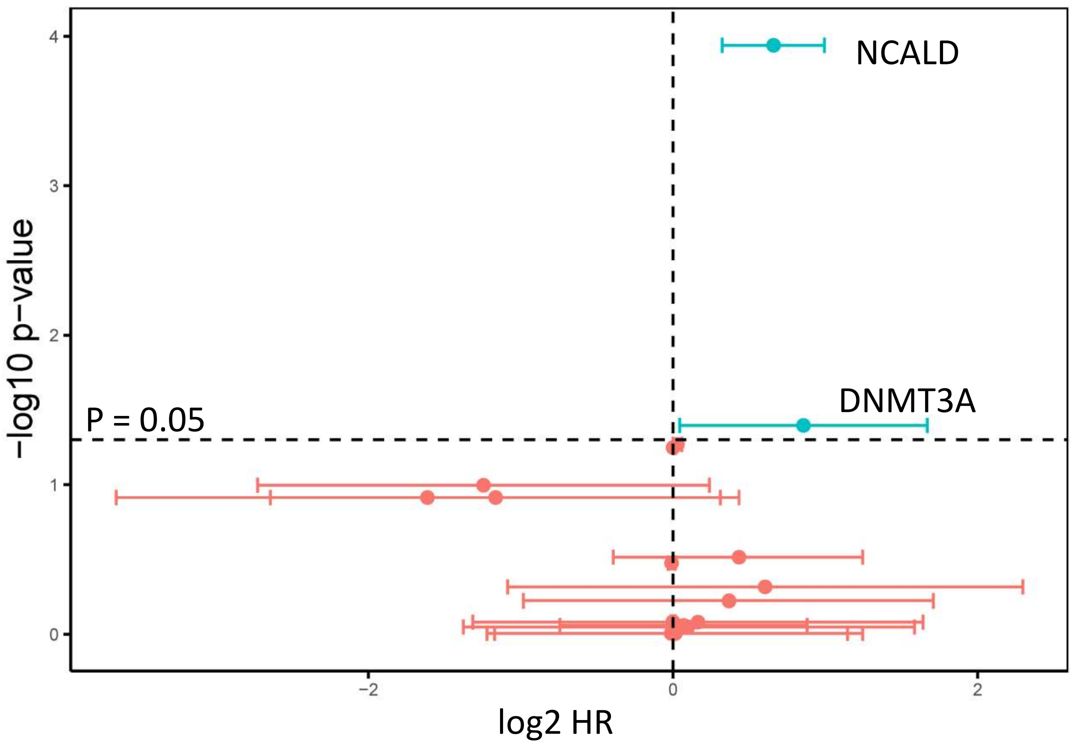

Additional file 2: Figure S6

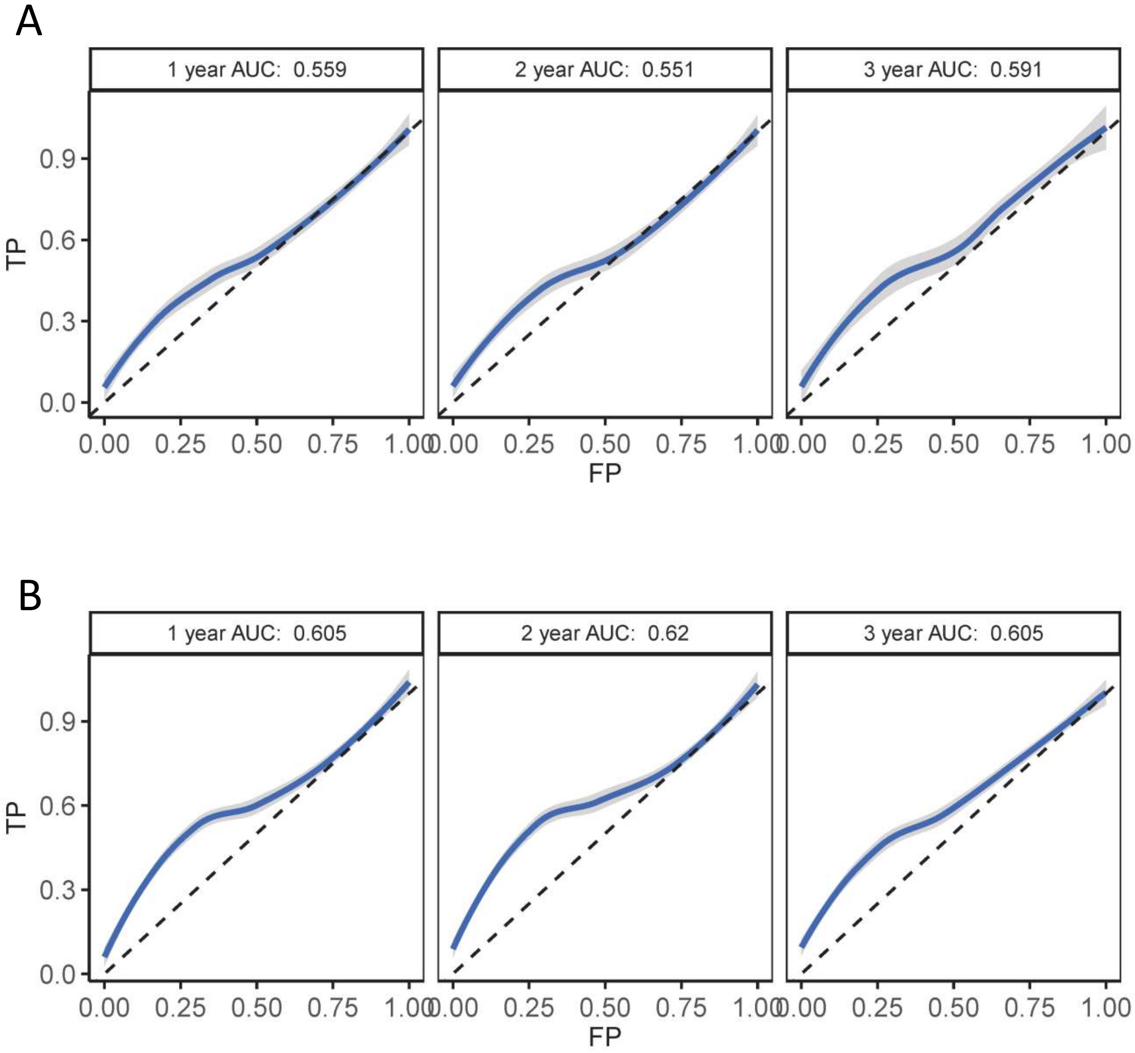

Additional file 2: Figure S7

A

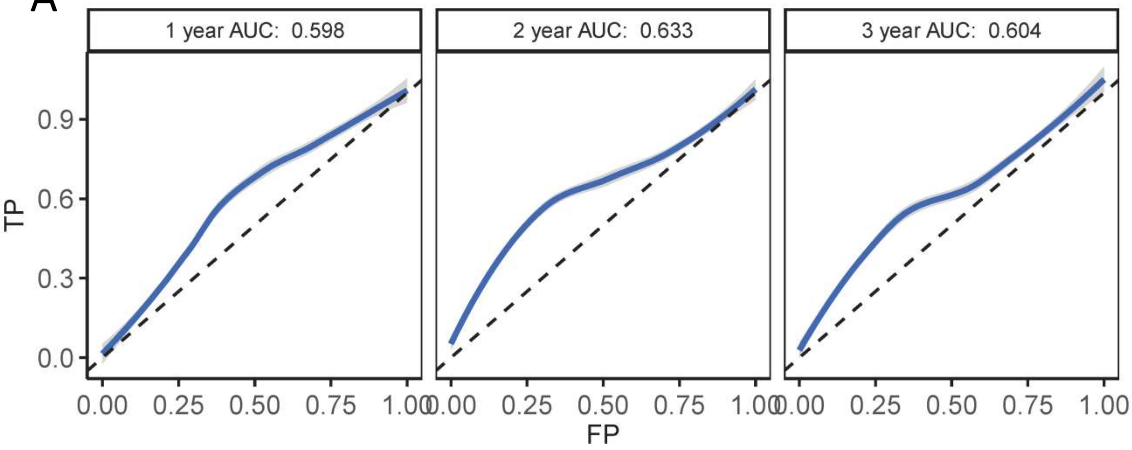

B

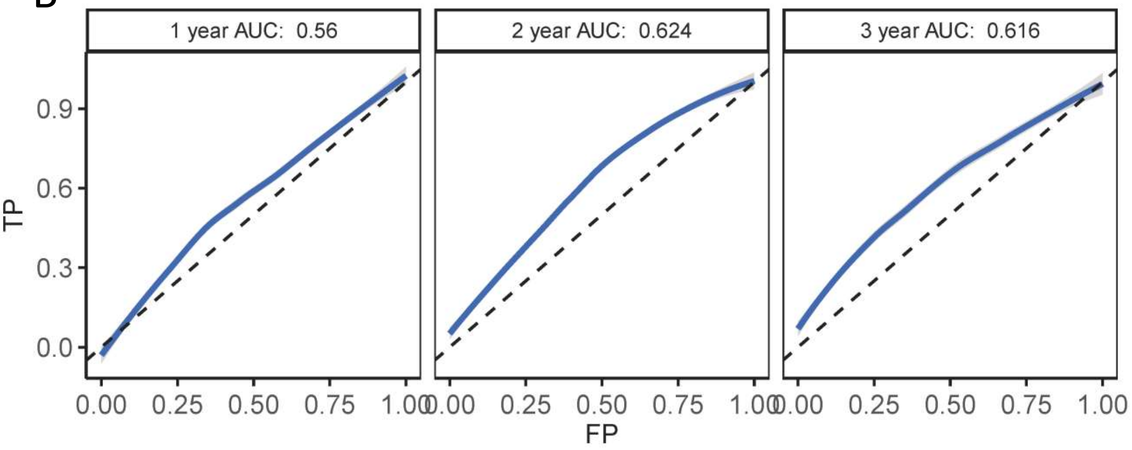

Additional file 2: Figure S8

A

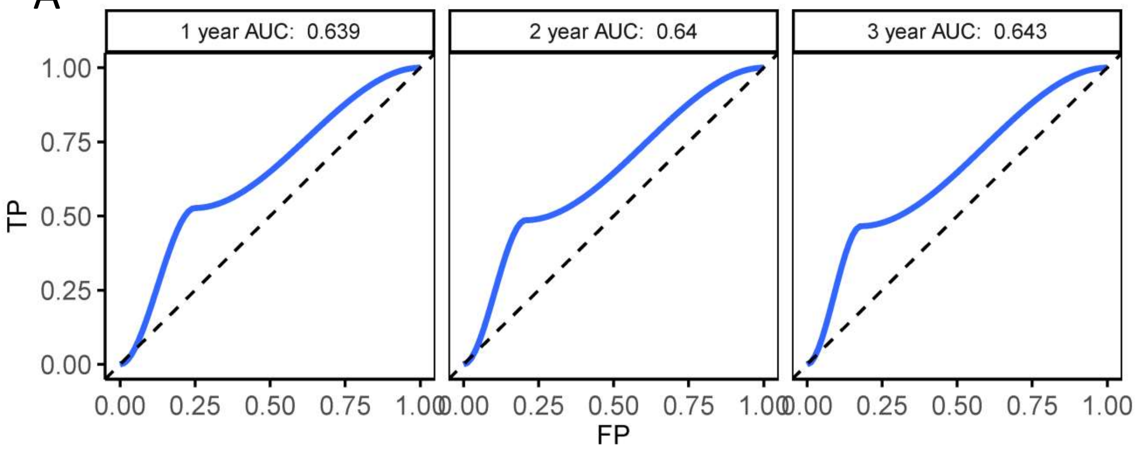

B

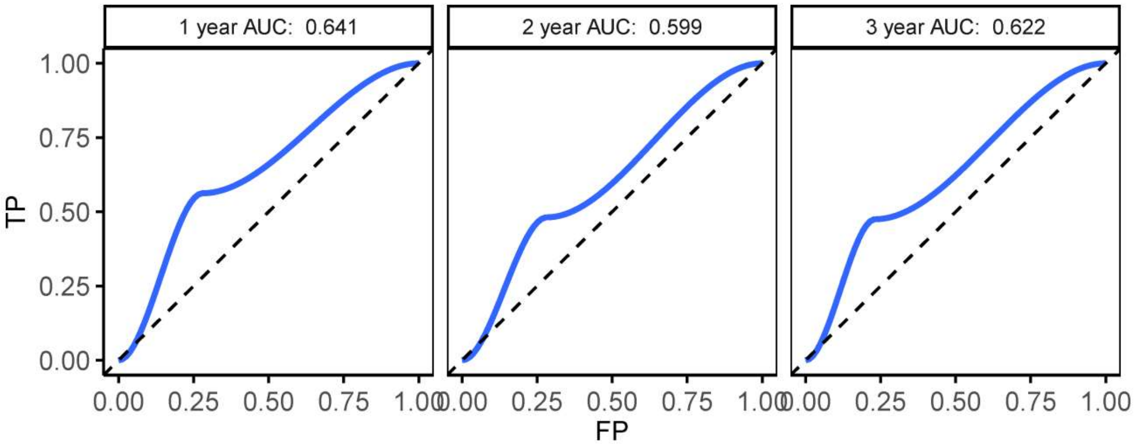

Additional file 2: Figure S9

A

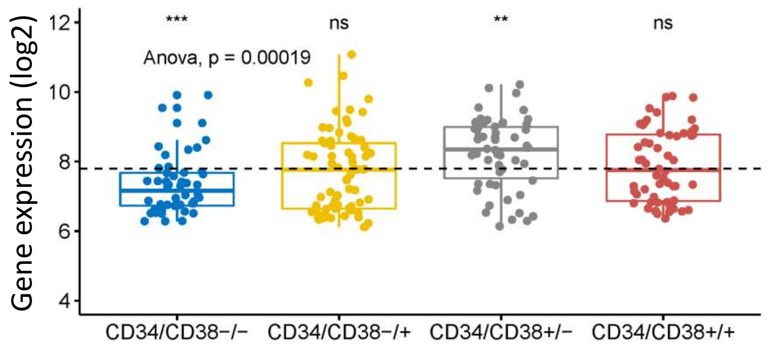

B

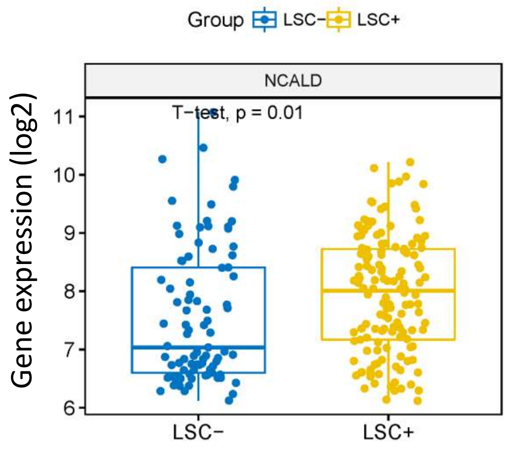

Supplement: Supplementary file 2 — Additional file 2: Figure S1. High NCALD expression predicts worse survival of CN-AML. The X axis represents time (months) and the Y axis represents survival probability. Kaplan-Meier curves were used for OS in different NCALD expression groups of CN-AML patients from GSE71014. The cutoff values for the high and low NCALD groups were 6.8281. OS, P < 0.0001; log rank test. Figure S2. High NCALD expression predicts poor survival of CN-AML. The X axis represents time and the Y axis represents survival probability. All patients were divided into two groups based on quartiles of NCALD expression levels. Top 75% patients are NCALD high expression group and the other 25% patients are NCALD low expression group by ranking NCALD gene expression from high to low. A, Kaplan-Meier curves were used for EFS and OS in different NCALD expression groups of CN-AML in the TCGA dataset. EFS, P < 0.0001; OS, P = 0.00035; log rank test. B, Kaplan-Meier curves were used for OS in different NCALD expression groups of CN-AML in GSE12417. OS, P < 0.0001; log rank test. C, Kaplan-Meier curves were used for OS in different NCALD expression groups in GSE22778. OS, P < 0.0001; log rank test. Figure S3. High NCALD expression predicts poor survival of CN-AML. The X axis represents time (months) and the Y axis represents survival probability. All patients were divided into two groups based on quartiles of NCALD expression levels. Top 75% patients are NCALD high expression group and the other 25% patients are NCALD low expression group by ranking NCALD gene expression from high to low. Kaplan-Meier curves were used for OS in different NCALD expression groups of CN-AML patients from GSE71014. OS, P < 0.0001; log rank test. Figure S4. High NCALD expression predicts poor survival of AML patients after allo-HSCT or chemotherapy from the TCGA dataset. The X axis represents time (months) and the Y axis represents survival probability. All patients were divided into two groups based on quartiles of NCALD e [file 12967_2019_1904_MOESM2_ESM.pdf]
